# Cold Weather Injuries Among the Active and Reserve Components of the U.S. Armed Forces, July 2020–June 2025

**Published:** 2026-02-04

## Abstract

From July 2024 through June 2025, a total of 806 members of the active (n=702) and reserve (n=104) components of the U.S. Armed Forces had at least 1 cold weather injury. Compared to the 2023-2024 cold season, the cold weather injury rates during the 2024-2025 cold season increased by 41.8% (from 38.6 to 54.7 per 100,000 person-years) and 45.8% (from 8.5 to 12.4 per 100,000 person-years) in the active and reserve components, respectively. The Army, Navy, and Marine Corps recorded their highest cold weather injury rates during the 2024-2025 season of the 5-year surveillance period. Frostbite was the most common cold weather injury in the Army, Navy, and Marine Corps, with the Marine Corps experiencing the largest surge in frostbite rates. Over the entire surveillance period, U.S. active component service member cold weather injury rates were generally higher among male service members, non-Hispanic Black individuals, and those under age 20 years.

**What are the new findings?**

The incidence rate of cold weather injuries among active component service members increased by over 40% between the 2023-2024 and 2024-2025 cold seasons, resulting in a 5-year rate of 41.5 per 100,000 person-years. This increase was primarily attributable to higher rates in the Army, Navy, and Marine Corps. The Marine Corps evinced the largest incidence rate increase (77.4%) during the 2024-2025 cold season. This year's update expanded cold injury surveillance to include “other specified and unspecified effects of reduced temperature,” to provide a more comprehensive assessment of cold weather injuries.

**What is the impact on readiness and force health protection?**

Despite the terminology, cold weather injuries can occur in a variety of conditions, and in much warmer temperatures than expected, particularly during operations or training in wet or aquatic environments. It is essential that both service members and leadership understand the hazards in their environments, the risks to health, and proven prevention strategies, including weather-appropriate clothing, clean, dry socks and footwear, and proper protective gear for bodily extremities.

Cold weather injuries are of significant military concern due to potential effects on service members (e.g., morbidity and potential disability) and the total force (e.g., adverse impacts on operations and costs of treatment). ^1 , 2^ In response, the U.S. Armed Forces have developed, and are continually improving, their training, doctrine, procedures, and protective equipment and clothing to counter the threat of cold environments. ^3 - 6^ Although these measures are effective when properly implemented, cold weather injuries continue to affect hundreds of service members each cold season due to exposures to both cold and wet environments. ^7 , 8^

Cold weather injuries can be broadly categorized in 2 major groups: those with a central effect and those primarily affecting the body's periphery. Hypothermia occurs if the body cannot maintain a core temperature at or above 95°F. If skin temperatures reach 95°F, the body's physiological response is triggered to minimize loss of heat and maintain core temperature for vital organ protection. ^9 , 10^ This response is achieved by decreasing blood flow to the extremities and redistributing warm blood to the body's core. ^9 - 11^ Lack of blood flow to the extremities, even before a drop in core temperature, is the leading cause of peripheral cold injuries.

Initially, hypothermia may impair cognition (e.g., confusion, slurred speech, memory loss), heart rate, and breathing. Severe hypothermia can lead to loss of consciousness, pulmonary edema, coma, ventricular arrhythmias (including ventricular fibrillation), and asystole. ^10 , 12 , 13^ Freezing atmospheric temperatures are not required to produce hypothermia, particularly when water immersion is involved. Because heat loss occurs 2 to 5 times faster in water compared to air, core body temperature can start to drop in water temperatures as warm as 80°F. ^10^

Peripheral cold injuries, which mainly affect the hands, feet, and face, can be further classified as either freezing injuries, such as frostbite, or non-freezing injuries, such as immersion foot. Freezing peripheral injury is defined as the damage sustained by tissues when skin temperatures fall below freezing, most frequently affecting tissues of the ears, nose, cheeks, chin, fingers, and toes. ^10 , 11 , 14 - 16^ A substantial proportion of patients with peripheral frostbite experience permanent changes in micro-circulation and disruption of localized nerve functions (e.g., reduced sensation in affected area). ^15^ Although most frostbite damage is minor, severe injury may lead to impaired functioning and inability to perform occupational tasks due to hypersensitivity to cold, chronic ulceration, vasospasm, localized osteoarthritis, or chronic pain. ^11 , 15 , 17^

Non-freezing peripheral injury includes a spectrum of localized injuries to the soft tissues, nerves, and vasculature of distal extremities that result from prolonged exposure to wet, cold (generally 32–59°F) conditions; the injury process is generally slower in warmer water. ^10 , 11 , 14 , 18^ Although most non-freezing peripheral injuries involve feet, any body part can be affected by the condition, including hands. ^19^ When immersion foot injury occurs, the foot becomes hyperemic (i.e., increased blood flow), painful, and swollen with continuous exposure; progression to blistering, decreased blood flow, ulceration, and gangrene is gradual. ^11 , 18 , 20^

Environmental factors that increase risk of cold weather injury include specific geographic locations including high altitudes, prolonged outdoor exposure to temperatures 40°F and lower, wind speeds exceeding 5 miles per hour, wet conditions due to rain or snow, or submersion in cold water, in addition to lack of adequate shelter and clothing. ^19^ Situational factors that increase risk of immersion foot include immobility, wet socks, and constrictive footwear. ^20 - 22^ Individual risk factors vary and include prior cold weather injury, improper acclimatization, dehydration, fatigue, inadequate nutrition, alcohol use, smoking, medications that impair compensatory responses (e.g., oral anti-hyperglycemics, beta-blockers, general anesthetic agents), and chronic disease (e.g., peripheral vascular disease, diabetes). ^10 , 11 , 16 , 20 - 22^

Continuous surveillance of cold weather injuries is essential to understand the magnitude of risk they pose, inform prevention efforts, and remind leaders of the hazards of training and operating in wet and cold environments. Department of Defense guidelines for reportable medical events (RMEs) require reporting of cases of hypothermia, freezing peripheral injuries (e.g., frostbite), and non-freezing peripheral injuries (e.g., immersion injuries, chilblains). ^23^

Since 2004, *MSMR* has published annual updates on the incidence of cold weather injuries affecting U.S. Armed Force members for the 5 most recent cold seasons. ^24^ The timing of these annual updates is intended to call attention to the recurring risks of such injuries as winter approaches in the Northern Hemisphere, where most members of the U.S. Armed Forces are assigned. Following a period of more limited scope, this update restored expanded cold weather injury surveillance last reported in 2017. ^25^ The current report now includes—in addition to frostbite, immersion injury, and hypothermia—unspecified cold injuries with “other effects of reduced temperature” for more complete case ascertainment.

## Methods

This surveillance population included all individuals who served in the active or reserve components of the U.S. Armed Forces at any time during the surveillance period of July 1, 2020 through June 30, 2025. For analysis purposes, a cold season was defined as July 1 through June 30 intervals, to allow for complete representation of cold weather seasons with annual summaries and appropriate comparisons. Due to data availability that began in January 2023, Space Force service members were classified separately starting in the 2022-2023 cold season; previously they were classified as Air Force.

Records of cold weather injuries for freezing peripheral injuries (i.e., frost-bite), non-freezing peripheral injuries (i.e., immersion hand, foot injuries), hypothermia, and unspecified cold weather injuries were identified from 2 sources: 1) RMEs submitted to the Disease Reporting System internet (DRSi) and 2) diagnostic codes from inpatient and outpatient medical encounters in the Defense Medical Surveillance System and in-theater records from the Theater Medical Data Store (which maintains electronic records of medical encounters of deployed service members). A cold weather injury case was defined by the presence of an RME or 1 of any of the following qualifying International Classification of Diseases, 10th Revision (ICD-10) codes in the first diagnostic position of an encounter for frostbite (T33*, T34*), immersion injury (T69.0*), hypothermia (T68*), or other effects of reduced temperature (T69.8, T69.9). Additional analyses were conducted to examine the distribution of cold injury types by services to further assess trends.

To estimate the number of unique individuals who experienced a cold weather injury each cold season, and to avoid inclusion of follow-up health care encounters, only 1 cold weather injury per individual per season was included in the counts of ‘any cold weather injury’. For analyses of specific cold weather injury types (frostbite, immersion injury, hypothermia, unspecified), individuals could contribute a maximum of 1 case per cold weather injury type per season to the ‘all cold weather injuries’ count. For example, if an individual was diagnosed or reported with an immersion injury at 1 point during a cold season, then with frostbite later in the same cold season, each different injury type would be included in injury-specific calculations. If a service member had multiple medical encounters for the same cold weather injury, only 1 encounter was included in this analysis. Hospitalization encounters were prioritized over ambulatory health care visits.

Annual seasonal incidence rates (IRs) of cold weather injuries among active component service members (ACSMs) were calculated as incident cold weather injury diagnoses per 100,000 person-years (p-yrs) of service. Annual seasonal IRs of cold weather injuries among reservists were calculated as cases per 100,000 persons, using the total number of reserve component service members for each cold season of the surveillance period. Person counts were used as the denominator for reserve component because the lack of start and end dates for active duty service periods precluded accurate person-time calculation.

Cold weather injuries are summarized by the locations where service members were treated for those injuries, identified by a Defense Medical Information System Identifier (DMIS ID) of a health care encounter. Because such injuries can occur during field training, temporary duty, or outside usual duty stations, DMIS IDs were utilized as proxies for locations where cold weather injuries occurred.

## Results

### 2024–2025 cold season

**FIGURE 1.**
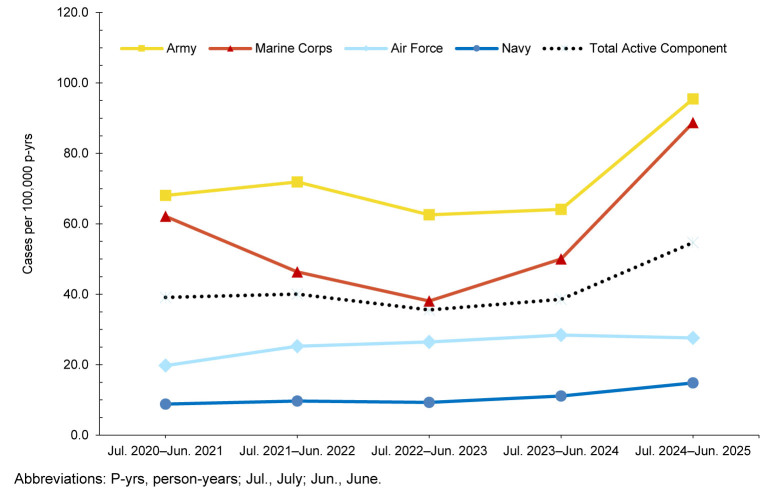
Annual Incidence Rates of Service Members Affected by Any Cold Injury (1 per person per year), by Service, Active Component, U.S. Armed Forces, July 2020–June 2025

**FIGURE 2.**
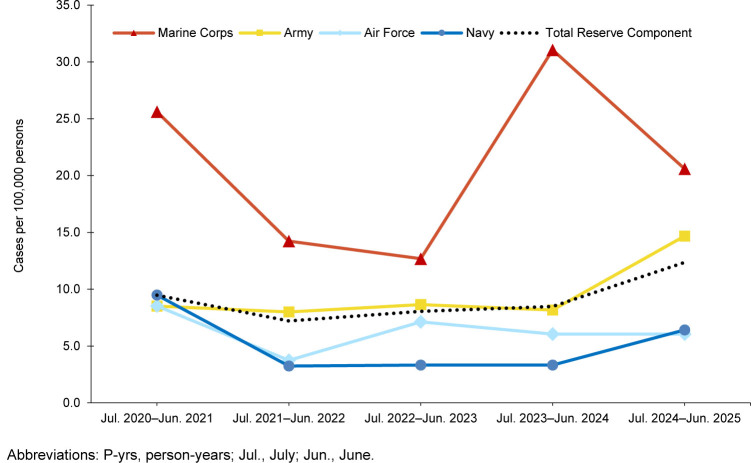
Annual Incidence Rates of Service Members Affected by Any Cold Injury (1 per person per year), by Service, Reserve Component, U.S. Armed Forces, July 2020–June 2025

From July 2024 through June 2025, a total of 806 members of the active (n=702) and reserve (n=104) components of the U.S. Armed Forces had at least 1 cold weather injury **(Table 1)** . In the active component, Army members had the highest rate of any cold weather injury (n=417, 95.5 per 100,000 p-yrs) during the 2024-2025 cold season, followed by members of the Marine Corps (n=147, 88.7 per 100,000 p-yrs), Air Force (n=85, 27.6 per 100,000 p-yrs), and Navy (n=48, 14.8 per 100,000 p-yrs). One active component Space Force member (10.6 per 100,000 p-yrs) and 4 active component Coast Guard members (10.0 per 100,000 p-yrs) were affected by cold weather injuries during the 2024-2025 cold season **(Table 1** , **Figure 1)** . Within the reserve component, Army personnel accounted for 77.9% of the cold injury cases (n=81, 14.7 per 100,000 persons) in the 2024-2025 cold season **(Table 1** , **Figure 2)** , although reservists in the Marine Corps (n=8, 20.6 per 100,000 persons) had higher rates of cold weather injuries.

**TABLE 1.** Annual Incidence of Service Members Affected by Any Cold Injury (1 per person per season), by Service and Component, July 2020–June 2025

		Army		Navy		Air Force		Marine Corps		Coast Guard		Space Force		All Services	
	No.	Rate ^a^	No.	Rate ^a^	No.	Rate ^a^	No.	Rate ^a^	No.	Rate ^a^	No.	Rate ^a^	No.	Rate ^a^
Active component														
All years (2020–2025)	1,649	72.2	178	10.7	405	25.4	490	56.9	22	11.1	4	17.1	2,748	41.5
Jul. 2020–Jun. 2021	325	68.1	30	8.8	65	19.7	112	62.2	3	7.4	0	0.0	535	39.1
Jul. 2021–Jun. 2022	339	71.9	33	9.7	82	25.3	82	46.4	6	14.9	0	0.0	542	40.0
Jul. 2022–Jun. 2023	284	62.6	31	9.3	84	26.4	65	38.1	4	10.3	1	20.6	469	35.6
Jul. 2023–Jun. 2024	284	64.1	36	11.1	89	28.4	84	50.0	5	12.8	2	22.2	500	38.6
Jul. 2024–Jun. 2025	417	95.5	48	14.8	85	27.6	147	88.7	4	10.0	1	10.6	702	54.7
Reserve component														
All years (2020–2025)	267		16		58		42		4		0		387	
Jul. 2020–Jun. 2021	49	8.5	6	9.5	16	8.5	11	25.6	1	15.1	0	0.0	83	9.5
Jul. 2021–Jun. 2022	45	8.0	2	3.2	7	3.7	6	14.2	2	30.3	0	0.0	62	7.2
Jul. 2022–Jun. 2023	47	8.7	2	3.3	13	7.1	5	12.7	0	0.0	0	0.0	67	8.1
Jul. 2023–Jun. 2024	45	8.2	2	3.3	11	6.1	12	31.1	1	15.1	0	0.0	71	8.5
Jul. 2024–Jun. 2025	81	14.7	4	6.4	11	6.1	8	20.6	0	0.0	0	0.0	104	12.4
Overall, active and reserve														
All years (2020–2025)	1,916		194		463		532		0		4		3,135	
Jul. 2020–Jun. 2021	374		36		81		123		0		0		618	
Jul. 2021–Jun. 2022	384		35		89		88		0		0		604	
Jul. 2022–Jun. 2023	331		33		97		70		0		1		536	
Jul. 2023–Jun. 2024	329		38		100		96		0		2		571	
Jul. 2024–Jun. 2025	498		52		96		155		0		1		806	

Abbreviations: No., number; Jul., July; Jun., June.

a Active component rate per 100,000 person-years; reserve component rate per 100,000 persons.

Frostbite was the most common type of cold weather injury among active component Army (n=167, 35.1%, **Table 2a** ), Marine Corps (n=63, 40.1%, **Table 2d** ) and Air Force (n=49, 53.3%, **Table 2c** ) members in 2024-2025, whereas immersion injury (n=15, 30.6%) and hypothermia (n=15, 30.6%) were the most common types of cold weather injuries among Navy service members **(Table 2b)** .

**TABLE 2a.** Annual Incidence of Frostbite, Immersion Injury, and Hypothermia Among All Cold Injuries (1 type per person per season), U.S. Army Active Component, July 2020–June 2025

		Frostbite		Immersion Injury		Hypothermia		Unspecified ^a^		All Cold Injuries	
	No.	Rate ^b^	No.	Rate ^b^	No.	Rate ^b^	No.	Rate ^b^	No.	Rate ^b^
Total	791	34.7	466	20.4	178	7.8	434	19.0	1,869	81.9
Sex										
Male	688	35.8	415	21.6	158	8.2	344	17.9	1,605	83.4
Female	103	28.7	51	14.2	20	5.6	90	25.1	264	73.7
Race and ethnicity										
White, non-Hispanic	283	23.9	221	18.6	77	6.5	149	12.6	730	61.6
Black, non-Hispanic	355	76.7	155	33.5	52	11.2	201	43.4	763	164.8
Hispanic	87	21.1	69	16.7	22	5.3	57	13.8	235	56.9
Other	66	29.8	21	9.5	27	12.2	27	12.2	141	63.7
Age, *y*										
<20	79	58.0	58	42.6	17	12.5	46	33.8	200	146.9
20–24	348	50.8	229	33.5	91	13.3	183	26.7	851	124.3
25–29	172	31.2	87	15.8	39	7.1	105	19.1	403	73.2
30–34	91	24.0	58	15.3	14	3.7	52	13.7	215	56.8
35–39	57	20.5	17	6.1	11	4.0	16	5.7	101	36.3
40–44	26	16.8	11	7.1	4	2.6	23	14.8	64	41.3
45+	18	18.2	6	6.1	2	2.0	9	9.1	35	35.3
Rank										
Recruit trainee	7	15.3	9	19.7	5	10.9	16	35.0	37	80.9
Enlisted	706	39.8	414	23.4	161	9.1	388	21.9	1,669	94.2
Officer	78	16.8	43	9.3	12	2.6	30	6.5	163	35.1
Military occupation										
Infantry, artillery, armor, combat engineering	354	62.7	234	41.4	94	16.7	135	23.9	817	144.7
Motor transport	33	48.3	10	14.6	3	4.4	23	33.7	69	101.0
Repair, engineering	102	23.1	63	14.3	23	5.2	67	15.2	255	57.8
Communications, intelligence	144	25.5	89	15.7	28	4.9	101	17.9	362	64.0
Health care	47	21.2	14	6.3	13	5.9	28	12.6	102	46.0
Other	111	26.4	56	13.3	17	4.0	80	19.0	264	62.7
Cold season (July–June)										
2020–2021	181	37.9	80	16.8	38	8.0	57	11.9	356	74.6
2021–2022	194	41.1	67	14.2	38	8.1	75	15.9	374	79.3
2022–2023	124	27.3	76	16.7	30	6.6	79	17.4	309	68.1
2023–2024	125	28.2	120	27.1	33	7.5	76	17.2	354	79.9
2024–2025	167	38.2	123	28.2	39	8.9	147	33.7	476	109.0

Abbreviations: No., number; *y* , years.

a Includes diagnoses for “other effects of reduced temperature.”

b Rate per 100,000 person-years.

**TABLE 2b.** Annual Incidence of Frostbite, Immersion Injury and Hypothermia Among All Cold Injuries (1 type per person per season), U.S. Navy Active Component, July 2020–June 2025

		Frostbite		Immersion Injury		Hypothermia		Unspecified ^a^		All Cold Injuries	
	No.	Rate ^b^	No.	Rate ^b^	No.	Rate ^b^	No.	Rate ^b^	No.	Rate ^b^
Total	59	3.6	38	2.3	58	3.5	25	1.5	180	10.8
Sex										
Male	49	3.7	36	2.7	49	3.7	17	1.3	151	11.5
Female	10	2.9	2	0.6	9	2.6	8	2.3	29	8.4
Race and ethnicity										
White, non-Hispanic	26	3.2	16	2.0	35	4.3	11	1.4	88	10.9
Black, non-Hispanic	12	4.5	8	3.0	8	3.0	4	1.5	32	11.9
Hispanic	12	4.0	9	3.0	8	2.7	4	1.3	33	11.1
Other	9	3.1	5	1.7	7	2.4	6	2.1	27	9.4
Age, *y*										
<20	7	7.2	9	9.3	8	8.3	6	6.2	30	31.0
20–24	16	3.3	14	2.9	28	5.7	9	1.8	67	13.7
25–29	15	3.8	6	1.5	17	4.3	3	0.8	41	10.3
30–34	6	2.1	2	0.7	4	1.4	2	0.7	14	4.9
35–39	8	3.7	2	0.9	1	0.5	3	1.4	14	6.5
40–44	4	3.5	3	2.6	0	0.0	1	0.9	8	7.0
45+	3	4.6	2	3.0	0	0.0	1	1.5	6	9.1
Rank										
Recruit trainee	2	7.7	1	3.9	0	0.0	0	0.0	3	11.6
Enlisted	45	3.3	33	2.4	54	4.0	23	1.7	155	11.4
Officer	12	4.3	4	1.4	4	1.4	2	0.7	22	7.9
Military occupation										
Infantry, artillery, armor, combat engineering	3	2.9	1	1.0	5	4.9	0	0.0	9	8.8
Motor transport	1	1.5	1	1.5	16	24.6	2	3.1	20	30.7
Repair, engineering	16	2.2	13	1.8	13	1.8	9	1.2	51	7.1
Communications, intelligence	5	1.9	8	3.0	3	1.1	3	1.1	19	7.2
Health care	17	10.0	0	0.0	4	2.4	5	3.0	26	15.3
Other	17	5.0	15	4.4	17	5.0	6	1.8	55	16.3
Cold season (July–June)										
2020–2021	9	2.6	3	0.9	13	3.8	5	1.5	30	8.8
2021–2022	12	3.5	8	2.3	13	3.8	1	0.3	34	10.0
2022–2023	8	2.4	7	2.1	12	3.6	4	1.2	31	9.3
2023–2024	20	6.2	5	1.5	5	1.5	6	1.8	36	11.1
2024–2025	10	3.1	15	4.6	15	4.6	9	2.8	49	15.1

Abbreviations: No., number; *y* , years.

a Includes diagnoses for “other effects of reduced temperature.”

b Rate per 100,000 person-years.

**TABLE 2c.** Annual Incidence of Frostbite, Immersion Injury and Hypothermia Among All Cold Injuries (1 type per person per season), U.S. Air Force Active Component, July 2020–June 2025

		Frostbite		Immersion Injury		Hypothermia		Unspecified ^a^		All Cold Injuries	
	No.	Rate ^b^	No.	Rate ^b^	No.	Rate ^b^	No.	Rate ^b^	No.	Rate ^b^
Total	243	15.3	34	2.1	47	3.0	123	7.7	447	28.1
Sex										
Male	214	17.1	31	2.5	36	2.9	94	7.5	375	30.0
Female	29	8.5	3	0.9	11	3.2	29	8.5	72	21.1
Race and ethnicity										
White, non-Hispanic	129	14.2	19	2.1	25	2.8	68	7.5	241	26.6
Black, non-Hispanic	48	21.6	5	2.3	5	2.3	21	9.5	79	35.6
Hispanic	38	14.0	5	1.8	9	3.3	20	7.4	72	26.6
Other	28	14.5	5	2.6	8	4.1	14	7.3	55	28.5
Age, *y*										
<20	17	25.1	3	4.4	4	5.9	12	17.7	36	53.1
20–24	117	26.4	16	3.6	17	3.8	49	11.1	199	44.9
25–29	45	11.3	6	1.5	13	3.3	22	5.5	86	21.6
30–34	30	10.0	1	0.3	9	3.0	20	6.7	60	20.0
35–39	22	9.3	7	3.0	1	0.4	13	5.5	43	18.2
40–44	8	7.6	0	0.0	3	2.9	6	5.7	17	16.2
45+	4	9.2	1	2.3	0	0.0	1	2.3	6	13.9
Rank										
Recruit trainee	0	0.0	0	0.0	1	4.8	2	9.7	3	14.5
Enlisted	221	17.5	27	2.1	35	2.8	109	8.6	392	31.0
Officer	22	7.2	7	2.3	11	3.6	12	3.9	52	16.9
Military occupation										
Infantry, artillery, armor, combat engineering	13	99.5	1	7.7	0	0.0	2	15.3	16	122.4
Motor transport	1	8.3	0	0.0	0	0.0	0	0.0	1	8.3
Repair, engineering	91	19.2	11	2.3	8	1.7	37	7.8	147	31.1
Communications, intelligence	40	11.8	4	1.2	6	1.8	19	5.6	69	20.4
Health care	9	6.2	2	1.4	3	2.1	13	8.9	27	18.5
Other	89	14.6	16	2.6	30	4.9	52	8.5	187	30.6
Cold season (July–June)										
2020–2021	46	14.0	1	0.3	9	2.7	15	4.6	71	21.6
2021–2022	53	16.3	5	1.5	6	1.8	30	9.2	94	29.0
2022–2023	48	15.1	7	2.2	7	2.2	28	8.8	90	28.3
2023–2024	47	15.0	10	3.2	13	4.2	30	9.6	100	31.9
2024–2025	49	15.9	11	3.6	12	3.9	20	6.5	92	29.9

Abbreviations: No., number; *y* , years.

a Includes diagnoses for “other effects of reduced temperature.”

b Rate per 100,000 person-years.

**TABLE 2d.** Annual Incidence of Frostbite, Immersion Injury and Hypothermia Among All Cold Injuries (1 type per person per season), U.S. Marines Active Component, July 2020–June 2025

		Frostbite		Immersion Injury		Hypothermia		Unspecified ^a^		All Cold Injuries	
	No.	Rate ^b^	No.	Rate ^b^	No.	Rate ^b^	No.	Rate ^b^	No.	Rate ^b^
Total	194	22.5	157	18.2	92	10.7	69	8.0	512	59.4
Sex										
Male	172	22.1	142	18.2	81	10.4	60	7.7	455	58.3
Female	22	27.1	15	18.5	11	13.5	9	11.1	57	70.2
Race and ethnicity										
White, non-Hispanic	88	18.6	93	19.7	43	9.1	28	5.9	252	53.4
Black, non-Hispanic	50	57.2	12	13.7	23	26.3	21	24.0	106	121.2
Hispanic	39	17.1	40	17.5	12	5.2	15	6.6	106	46.4
Other	17	23.2	12	16.4	14	19.1	5	6.8	48	65.5
Age, *y*										
<20	28	24.3	83	72.0	23	20.0	9	7.8	143	124.1
20–24	105	25.8	56	13.8	53	13.0	46	11.3	260	64.0
25–29	36	22.9	14	8.9	10	6.4	9	5.7	69	43.9
30–34	19	23.9	3	3.8	3	3.8	3	3.8	28	35.2
35–39	6	9.8	1	1.6	1	1.6	2	3.3	10	16.3
40–44	0	0.0	0	0.0	2	7.2	0	0.0	2	7.2
45+	0	0.0	0	0.0	0	0.0	0	0.0	0	0.0
Rank										
Recruit trainee	2	6.3	61	191.6	12	37.7	1	3.1	76	238.7
Enlisted	134	18.6	89	12.3	75	10.4	57	7.9	355	49.2
Officer	58	53.9	7	6.5	5	4.6	11	10.2	81	75.3
Military occupation										
Infantry, artillery, armor, combat engineering	92	51.5	19	10.6	29	16.2	27	15.1	167	93.6
Motor transport	5	12.0	2	4.8	1	2.4	3	7.2	11	26.5
Repair, engineering	8	3.9	9	4.4	10	4.9	6	2.9	33	16.1
Communications, intelligence	26	12.5	11	5.3	9	4.3	16	7.7	62	29.8
Health care	0	0.0	0	0.0	0	0.0	0	0.0	0	0.0
Other	63	27.6	116	50.7	43	18.8	17	7.4	239	104.5
Cold season (July–June)										
2020–2021	53	29.4	25	13.9	21	11.7	15	8.3	114	63.3
2021–2022	33	18.7	23	13.0	18	10.2	11	6.2	85	48.1
2022–2023	22	12.9	28	16.4	11	6.4	7	4.1	68	39.8
2023–2024	23	13.7	35	20.8	18	10.7	12	7.1	88	52.4
2024–2025	63	38.0	46	27.8	24	14.5	24	14.5	157	94.8

Abbreviations: No., number; *y* , years.

a Includes diagnoses for “other effects of reduced temperature.”

b Rate per 100,000 person-years.

### Five cold seasons: July 2020–June 2025

The crude IR for all 5 cold seasons of any cold weather injury was 41.5 per 100,000 p-yrs for all ACSMs **(Table 1)** . In the most recent cold season, 2024-2025, the crude IR of any cold weather injury for all ACSMs increased by 41.8%, from 38.6 per 100,000 p-yrs in 2023-2024 to 54.7 per 100,000 p-yrs in 2024-2025 **(Table 1)** , the highest value documented during the 5-year surveillance period. Similarly, the crude IR of any cold weather injury for the reserve component increased by 45.8% in 2024-2025 (from 8.5 to 12.4 per 100,000 persons) from the prior season. Throughout the surveillance period, cold weather injury rates remained consistently higher among ACSMs in the Army and Marine Corps **(Figure 1)** .

During the 5-year surveillance period, overall rates of all cold weather injuries in the active component were generally higher among service members who were male (except in the Marine Corps), non-Hispanic Black individuals, and among the 2 youngest age groups (ages <20 and 20-24 years) **(Tables 2a** – **2d)** . When specific types of cold injury were considered, male and non-Hispanic Black service members had higher rates of frostbite in comparison to other types of injury **(Tables 2a** – **2d)** . Among all cold weather injury cases reported within the active component during the 5-year period, the Marine Corps demon-strated the highest recruit cold weather injury rate (238.7 per 100,000 p-yrs). With the exception of the Marine Corps, enlisted personnel had higher rates of cold weather injury compared to officers **(Tables 2a** – **2f)** .

**TABLE 2e.** Annual Incidence of Frostbite, Immersion Injury and Hypothermia Among All Cold Injuries (1 type per person per season), U.S. Coast Guard Active Component, July 2020–June 2025

		Frostbite		Immersion Injury		Hypothermia		Unspecified ^a^		All Cold Injuries	
	No.	Rate ^b^	No.	Rate ^b^	No.	Rate ^b^	No.	Rate ^b^	No.	Rate ^b^
Total	7	3.5	0	0.0	6	3.0	10	5.0	23	11.6
Sex										
Male	7	4.2	0	0.0	5	3.0	10	6.0	22	13.2
Female	0	0.0	0	0.0	1	3.2	0	0.0	1	3.2
Race and ethnicity										
White, non-Hispanic	5	4.0	0	0.0	3	2.4	8	6.4	16	12.8
Black, non-Hispanic	0	0.0	0	0.0	3	29.4	0	0.0	3	29.4
Hispanic	1	3.1	0	0.0	0	0.0	1	3.1	2	6.3
Other	1	3.2	0	0.0	0	0.0	1	3.2	2	6.4
Age, *y*										
<20	0	0.0	0	0.0	0	0.0	0	0.0	0	0.0
20–24	1	2.4	0	0.0	2	4.8	3	7.2	6	14.4
25–29	2	4.9	0	0.0	2	4.9	3	7.4	7	17.3
30–34	1	2.7	0	0.0	1	2.7	0	0.0	2	5.5
35–39	1	2.7	0	0.0	1	2.7	2	5.4	4	10.8
40–44	2	8.7	0	0.0	0	0.0	1	4.3	3	13.0
45+	0	0.0	0	0.0	0	0.0	1	8.4	1	8.4
Rank										
Recruit trainee	0	0.0	0	0.0	0	0.0	0	0.0	0	0.0
Enlisted	6	3.9	0	0.0	6	3.9	7	4.6	19	12.5
Officer	1	2.3	0	0.0	0	0.0	3	6.8	4	9.0
Military occupation										
Infantry, artillery, armor, combat engineering	0	0.0	0	0.0	0	0.0	0	0.0	0	0.0
Motor transport	1	3.2	0	0.0	2	6.3	2	6.3	5	15.8
Repair, engineering	2	3.3	0	0.0	1	1.6	2	3.3	5	8.1
Communications, intelligence	1	3.3	0	0.0	2	6.7	0	0.0	3	10.0
Health care	0	0.0	0	0.0	1	26.6	0	0.0	1	26.6
Other	3	4.2	0	0.0	0	0.0	6	8.4	9	12.6
Cold season (July–June)										
2020–2021	1	2.5	0	0.0	1	2.5	1	2.5	3	7.4
2021–2022	1	2.5	0	0.0	2	5.0	4	9.9	7	17.4
2022–2023	2	5.1	0	0.0	1	2.6	1	2.6	4	10.3
2023–2024	2	5.1	0	0.0	2	5.1	1	2.6	5	12.8
2024–2025	1	2.5	0	0.0	0	0.0	3	7.5	4	10.0

Abbreviations: No., number; *y* , years.

a Includes diagnoses for “other effects of reduced temperature.”

b Rate per 100,000 person-years.

**TABLE 2f.** Annual Incidence of Frostbite, Immersion Injury and Hypothermia Among All Cold Injuries (1 type per person per season), U.S. Space Force Active Component, July 2020–June 2025

		Frostbite		Immersion Injury		Hypothermia		Unspecified ^a^		All Cold Injuries	
	No.	Rate ^b^	No.	Rate ^b^	No.	Rate ^b^	No.	Rate ^b^	No.	Rate ^b^
Total	2	8.6	0	0.0	0	0.0	3	12.9	5	21.4
Sex										
Male	2	10.6	0	0.0	0	0.0	3	15.9	5	26.4
Female	0	0.0	0	0.0	0	0.0	0	0.0	0	0.0
Race and ethnicity										
White, non-Hispanic	1	6.9	0	0.0	0	0.0	0	0.0	1	6.9
Black, non-Hispanic	0	0.0	0	0.0	0	0.0	2	112.1	2	112.1
Hispanic	1	29.0	0	0.0	0	0.0	1	29.0	2	57.9
Other	0	0.0	0	0.0	0	0.0	0	0.0	0	0.0
Age, *y*										
<20	0	0.0	0	0.0	0	0.0	0	0.0	0	0.0
20–24	2	46.1	0	0.0	0	0.0	1	23.1	3	69.2
25–29	0	0.0	0	0.0	0	0.0	1	17.7	1	17.7
30–34	0	0.0	0	0.0	0	0.0	1	20.8	1	20.8
35–39	0	0.0	0	0.0	0	0.0	0	0.0	0	0.0
40–44	0	0.0	0	0.0	0	0.0	0	0.0	0	0.0
45+	0	0.0	0	0.0	0	0.0	0	0.0	0	0.0
Rank										
Recruit trainee	0	0.0	0	0.0	0	0.0	0	0.0	0	0.0
Enlisted	2	16.8	0	0.0	0	0.0	2	16.8	4	33.6
Officer	0	0.0	0	0.0	0	0.0	1	8.7	1	8.7
Military occupation										
Infantry, artillery, armor, combat engineering	0	0.0	0	0.0	0	0.0	0	0.0	0	0.0
Motor transport	0	0.0	0	0.0	0	0.0	0	0.0	0	0.0
Repair, engineering	0	0.0	0	0.0	0	0.0	0	0.0	0	0.0
Communications, intelligence	0	0.0	0	0.0	0	0.0	0	0.0	0	0.0
Health care	0	0.0	0	0.0	0	0.0	0	0.0	0	0.0
Other	2	9.5	0	0.0	0	0.0	3	14.2	5	23.7
Cold season (July–June)										
2020–2021	0	0.0	0	0.0	0	0.0	0	0.0	0	0.0
2021–2022	0	0.0	0	0.0	0	0.0	0	0.0	0	0.0
2022–2023	1	20.6	0	0.0	0	0.0	0	0.0	1	20.6
2023–2024	1	11.1	0	0.0	0	0.0	2	22.2	3	33.3
2024–2025	0	0.0	0	0.0	0	0.0	1	10.6	1	10.6

Abbreviations: No., number; *y* , years.

a Includes diagnoses for “other effects of reduced temperature.”

b Rate per 100,000 person-years.

Throughout the 5-year surveillance period, a total of 38 ACSMs (1.4% of total) were hospitalized. The Army (n=25) and Marine Corps (n=8) accounted for the majority (86.8%) of hospitalized cases (data not shown).

### Patterns and trends in service branches

### Army

Within the Army active component, total cold injury cases and IRs increased from 356 cases (74.6 per 100,000) in 2020-2021 to 476 cases (109.0 per 100,000) in 2024-2025, representing a 46.1% increase during the surveillance period **(Table 2a** , **Figure 3a)** . Frostbite was the most common cold injury type overall, with rates increasing by 35.4% in 2024-2025 compared to the prior season. Army IRs increased most for unspecified injuries, with values nearly tripling over the surveillance period (from 11.9 to 33.7 per 100,000 p-yrs). Increases in immersion injuries and hypothermia were slight-to-moderate and less pronounced.

**FIGURE 3a.**
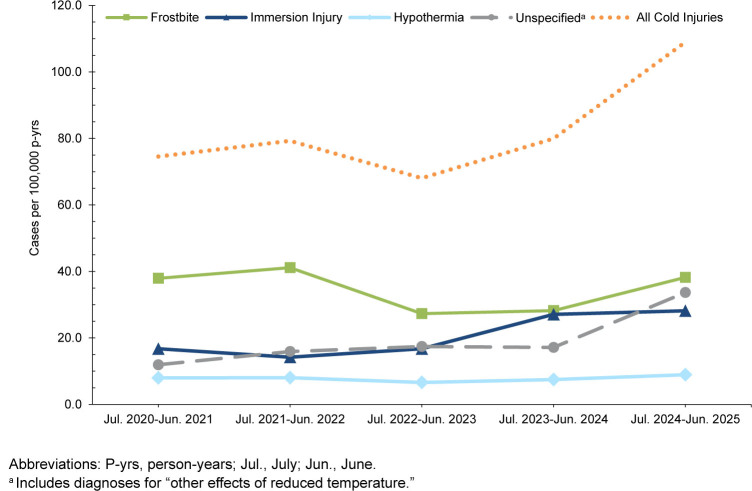
Annual Incidence Rates by Cold Injury Type Among Army Service Members, Active Component, U.S. Armed Forces, July 2020–June 2025

### Navy

Among the Navy active component, total cases and IRs increased from 30 cases (8.8 per 100,000 p-yrs) in 2020-2021 to 49 (15.1 per 100,000 p-yrs) in 2024-2025, representing a 71.6% IR increase over the surveillance period **(Table 2b** , **Figure 3b)** . The overall increase for the Navy was primarily driven by comparatively sharp rises in immersion injuries and hypothermia cases in 2024-2025, compared to prior seasons. The highest IR for the Navy during the 5-year surveillance period was seen for frostbite cases, followed closely by hypothermia. Counts and IRs of unspecified injuries were relatively lower and fluctuated over the surveillance period.

**FIGURE 3b.**
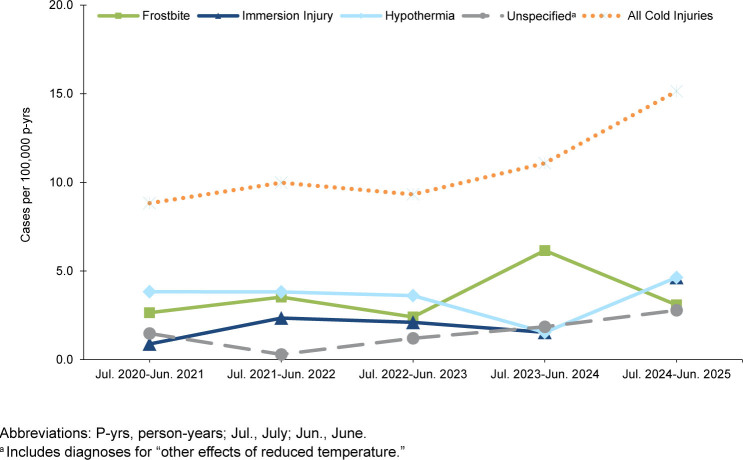
Annual Incidence Rates by Cold Injury Type Among Navy Service Members, Active Component, U.S. Armed Forces, July 2020–June 2025

### Air Force

Within the Air Force active component, total cold injury cases and IRs increased from 71 cases (21.6 per 100,000 p-yrs) in 2020-2021 to 92 (29.9 per 100,000 p-yrs) in 2024-2025 (38.4% IR increase), with the apex (100 cases, 31.9 per 100,000 p-yrs) during the 2023-2024 cold season **(Table 2c** , **Figure 3c)** . The observed Air Force increase was largely attributable to rises in immersion injuries and hypothermia cases. Rates of unspecified injuries, the second most common cold injury following frostbite, fluctuated throughout the surveillance period.

**FIGURE 3c.**
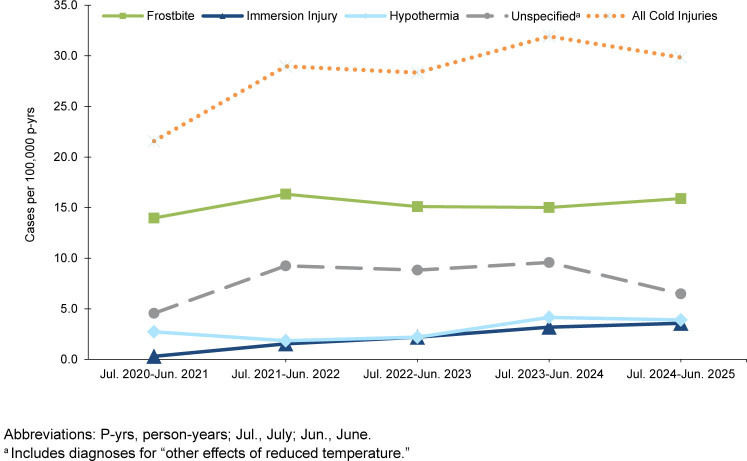
Annual Incidence Rates by Cold Injury Type Among Air Force Service Members, Active Component, U.S. Armed Forces, July 2020–June 2025

### Marine Corps

Among the Marine Corps active component, total cold injury cases and IRs increased from 114 cases (63.3 per 100,000 p-yrs) in 2020-2021 to 157 cases (94.8 per 100,000 p-yrs) in 2024-2025, representing a 49.8% increase during the surveillance period **(Table 2d** , **Figure 3d)** . Frostbite was the dominant cold injury, in both counts and IRs. Frostbite IRs in the Marine Corps nearly tripled during the most recent cold season compared to the prior season (38.0 per 100,000 p-yrs in 2024-2025 vs. 13.7 in 2023-2024). Immersion injuries and hypothermia also showed notable increases over time within the Marine Corps, while unspecified injuries, although smaller in magnitude, also rose sharply in 2024-2025 cold season.

**FIGURE 3d.**
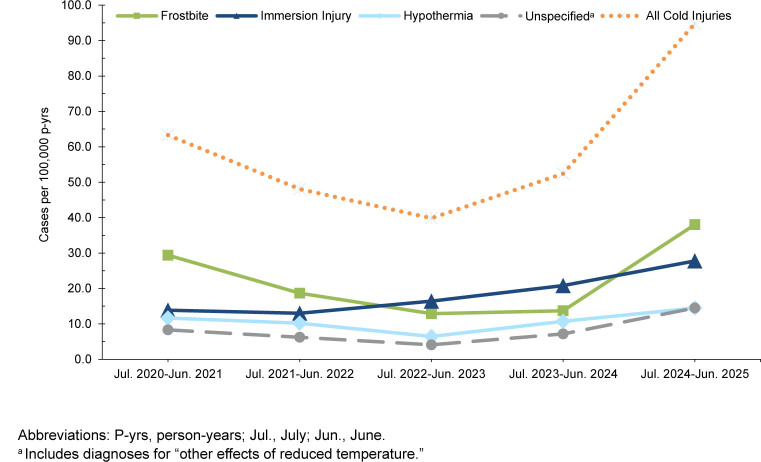
Annual Incidence Rates by Cold Injury Type Among Marine Corps Service Members, Active Component, U.S. Armed Forces, July 2020–June 2025

### Deployment-related cold weather injuries

During the 5-year surveillance period, a total of 82 cold weather injuries were diagnosed among service members deployed outside the U.S. (data not shown), of which 35 (42.7%) were frostbite, 33 (40.2%) were immersion injuries, 12 (14.6%) were hypothermia, and 2 (2.4%) were unspecified. Among the 28 cases of the 82 total deployment-associated cold weather injuries diagnosed during the 2024-2025 cold season, 17 were frostbite, 7 were immersion injuries, and 4 were hypothermia cases.

### Geographic locations of cold weather injuries

**FIGURE 4.**
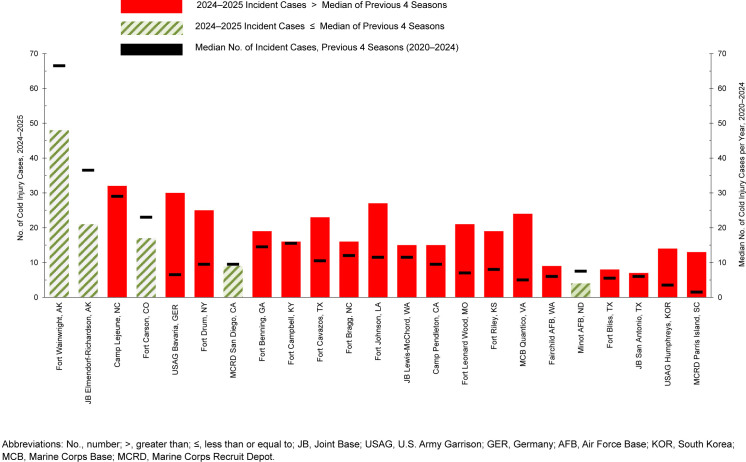
Annual Frequency (cold season 2024–2025) and Median Numbers (cold seasons 2020–2024) of Cold Injuries at Locations with at Least 25 Cold Injuries During the Surveillance Period, Active Component, U.S. Armed Forces, July 2020–June 2025

During the 5-year surveillance period, 23 military locations reported at least 25 incidents of cold weather injury (1 per person per cold season) among ACSMs. **Figure 4** charts the 2024-2025 seasonal numbers of cold weather injuries (1 per person per year) for each of those 23 locations, in addition to the median case numbers for the previous 4 cold seasons. The highest 5-year counts of incident cold weather injuries for seasons 2020 through 2025 were recorded at Fort Wainwright, Arkansas (n=335), Joint Base Elmendorf-Richardson, Arkansas (n=209), Marine Corps Base Camp Lejeune, North Carolina (n=115), Fort Carson, Colorado (n=104), and U.S. Army Garrison Bavaria, Germany (n=85) (data not shown).

## Discussion

Overall rates peaked in 2024-2025 for any cold weather injury among the U.S. active and reserve components, increasing by 41.8% and 45.8%, respectively, from the 2023-2024 season. During the 5-year surveillance period, the active components of all services experienced increased IRs for cold injuries. During the 2024-2025 cold season, the Army, Navy, and Marine Corps active components experienced their highest rates of any cold weather injury for the entire 5-year surveillance period. The Coast Guard and Space Force average less than 5 cases per year among their ACSMs, thus, small changes in the numbers of cases annually will result in abnormally large fluctuations in the injury rate. Frostbite was the most common cold weather injury in the Army, Marine Corps, and Navy, while the Marine Corps saw the largest surge in frostbite rates. In contrast, immersion injuries and hypothermia were the main causes of increases in the Navy and Air Force. Rates of unspecified cold injuries also increased substantially within the Army and Marine Corps, but remained lower and more variable in the Navy and Air Force.

The simultaneous increase in both specific and unspecified case rates suggests true increases in cold weather injury occurrence in the 2024-2025 cold season. The increase in IRs could indicate heightened exposure to environmental risk factors. The long-term complications of non-freezing injuries are similar to, and equally debilitating as, those produced by frostbite: hypersensitivity to cold, chronic pain, and severe pain induced by walking. ^17 , 18 , 20^

Similar to previous *MSMR* reports, the highest cold weather injury rates were observed among service members who were male, those in younger age groups, and non-Hispanic Black individuals. ^8 , 24^ Increasing rates of cold weather injury have also been noted among service members in the United Kingdom (U.K.) military with similar demographic characteristics. ^21 , 26 , 27^ Differences in physiological responses to cold stress have been observed between various racial and ethnic groups, with individuals of African descent demonstrating greater vasoconstriction responses compared to individuals of Asian or Caucasian descent. ^10 , 15 , 28^ Signs and symptoms of cold weather injury (e.g., skin redness, blotchy skin) may initially be more difficult to see on service members with skin of darker color. ^29 , 30^ Service members, leadership, and medical personnel should be educated on the early signs and symptoms of cold weather injuries for a wide range of skin types.

When examining the demographic groups with increased rates within the services, it should be noted that there were differences in the most frequently observed cold weather injury types. Younger marines had higher rates of immersion injuries, while younger soldiers had higher rates of frostbite. Such differences could indicate different situational risk factors, such as specific training activities, occupational tasks, and geographic regions, for cold weather injury among the service branches. A study of U.K. service personnel noted that the most common situational risk factors for non-freezing peripheral injury were standing guard, as well as wet socks and boots. ^21^ Unit leaders must be able to assess environmental, situational, and individual risk factors of their training and operational environments and understand how those factors increase risk of cold weather injuries for service members in their charge.

This analysis of cold weather injuries was unable to distinguish between injuries sustained during official military duties (e.g., training or operations) and those associated with unrelated or personal activities. This report expanded the scope of cold injuries beyond specified conditions (e.g., frostbite, immersion injury, hypothermia) to include “other specified and unspecified effects of reduced temperature.” That change contributed to an increased overall case count compared to last year's report. The increase in cold injury IRs was observed uniformly for all services and specific injury types, suggesting a genuine rise in cold injury incidence rather than solely an artifact of broadened inclusion criteria.

Cold weather injuries can be prevented by ensuring proper clothing, including layers that can be added or removed according to environmental conditions and specific physical activities, along with footwear that is non-constrictive, dry, and regularly changed if wet. ^9 , 10 , 22^ Proper hydration and nutrition, avoidance of long periods of sedentary or immobile positions, and planning for appropriate shelter and opportunities for re-warming are also important.

Military training or mission requirements in cold and wet weather conditions can preclude immediate warm or dry shelter, ability to change wet or damp clothing, or even healthy physical activity. ^2 , 3 , 11^ To prepare for all circumstances posing a threat for cold weather injury, service members should be cognizant of, and able to identify, signs of cold weather injury in addition to environmental, individual, and situational risk factors. Service members should also be aware of protective measures for themselves and their fellow service members, whether during training, operations, combat, or recreational activities in wet or freezing conditions.
